# Contrastive learning–guided multi-meta attention network for breast ultrasound video diagnosis

**DOI:** 10.3389/fonc.2022.952457

**Published:** 2022-10-24

**Authors:** Xiaoyang Huang, Zhi Lin, Shaohui Huang, Fu Lee Wang, Moon-Tong Chan, Liansheng Wang

**Affiliations:** ^1^ Department of Computer Science, School of Informatics, Xiamen University, Xiamen, China; ^2^ School of Science and Technology, Hong Kong Metropolitan University, Hong Kong, Hong Kong SAR, China

**Keywords:** ultrasound sequence, video classification, breast lesion, contrastive learning, multi-meta attention network

## Abstract

Breast cancer is the most common cause of cancer death in women. Early screening and treatment can effectively improve the success rate of treatment. Ultrasound imaging technology, as the preferred modality for breast cancer screening, provides an essential reference for early diagnosis. Existing computer-aided ultrasound imaging diagnostic techniques mainly rely on the selected key frames for breast cancer lesion diagnosis. In this paper, we first collected and annotated a dataset of ultrasound video sequences of 268 cases of breast lesions. Moreover, we propose a contrastive learning–guided multi-meta attention network (CLMAN) by combining a deformed feature extraction module and a multi-meta attention module to address breast lesion diagnosis in ultrasound sequence. The proposed feature extraction module can autonomously acquire key information of the feature map in the spatial dimension, whereas the designed multi-meta attention module is dedicated to effective information aggregation in the temporal dimension. In addition, we utilize a contrast learning strategy to alleviate the problem of high imaging variability within ultrasound lesion videos. The experimental results on our collected dataset show that our CLMAN significantly outperforms existing advanced methods for video classification.

## 1 Introduction

According to the World Cancer Report (1), the number of new cases of breast cancer has reached 2.26 million worldwide in 2020, and breast cancer accounts for about 6.9% of all deaths from cancer worldwide, ranking fifth. Early detection and timely treatment can effectively improve the survival prognosis of breast cancer patients, prolong their survival years, and improve the people’s living standards. Because it is non-invasive, inexpensive, safe, and free of ionizing radiation, ultrasound imaging is currently the most commonly used technique for the early detection of breast lesions. However, ultrasound imaging provides low-quality imaging, mainly because interference from the ultrasound reflective wavefront causes speckle noise on imaging. During the acquisition or examination of a breast sequence, the operator usually needs to apply processing such as filtering, adjusting brightness levels, and scaling the image to improve the quality of ultrasound imaging, whereas interpreting ultrasound imaging usually requires an experienced and well-trained radiologist. However, in some cases, the breast lesion in the ultrasound imaging is ambiguous, and even experienced radiologists are unable to accurately determine its benignity or malignancy [in medical practice, BI-RADS 1–3 are usually considered benign, BI-RADS 4 for suspicious malignant, and BI-RADS 5–6 for malignant (2)].

Computer-assisted technology has provided new ideas for the diagnosis of breast lesions by ultrasound imaging. With the help of computer-aided diagnosis, the operation-dependent impact of ultrasound imaging can be minimized. At the same time, computer-aided diagnosis can also reduce the workload of radiologists. Most of the existing computer-aided diagnostic techniques analyze a single frame (key frames) in the video sequence of pathology acquisition. Although it helps to reduce the computer diagnostic time, it also reveals two significant problems: first, it is challenging to select typical key frames representing pathology samples; second, too much pathology diagnostic information is lost in the video sequence. The field of benign and malignant classification for breast lesions by ultrasound video sequences is in urgent need of research.

Therefore, we propose an automatic diagnosis model for ultrasound sequences, which uses deep learning methods to achieve high accuracy in classification recognition to assist medical diagnosis tasks. The designed diagnostic model weighs spatial dimensional information through the non-local module, on the one hand, and adaptive and fine-grained attention weight scoring for each feature dimension of each frame through the multi-meta attention module, on the other hand, focusing on the key information in the samples in a self-learning manner. This approach can accept samples of different sequence lengths and make full use of the potential connections between frames in the sample by weighting and aggregating the features of each frame through the aggregation module to improve the accuracy of diagnosis.

The contributions of this work can be summarized as follows: a) We develop a new network for learning video-level classification of breast lesions. b) We collected an ultrasound video dataset (268 sequences) for breast lesion classification. c) A deformed feature extraction module is proposed to facilitate high-quality deep feature representation, whereas a multi-meta attention module is developed to acquire key feature information at the video level adaptively. d) The experimental results show that our network achieves a new state-of-the-art performance in the breast ultrasound lesion classification task on our collected dataset.

## 2 Related work

### 2.1 Breast ultrasound classification

Classification of breast lesion pathology is a primary task in computer-aided diagnosis projects. Researchers working on breast ultrasound-related topics have proposed a number of effective deep learning schemes. Han et al. (3) used deep convolutional networks pre-trained on grayscale nature images to discriminate between benign and malignant. Although the lesion regions of interest used in this scheme were all provided by radiologists, this study demonstrated that breast lesion features extracted by deep learning–based networks can achieve comparable classification performance to hand-designed feature methods. To further avoid the potential missing effects that result from manual intervention in the region of interest selection, Cheng et al. (4) proposed the utilization of an unsupervised stacked denoising auto-encoder to extract high-level feature representations for breast lesion imaging with supervised fine-tuning training. Diagnosis models constructed in a deep learning manner usually require a large amount of training data to achieve significant classification results. However, because most cases are benign, the imbalance of medical data makes it particularly difficult to collect sufficient training samples. To alleviate the problem of model underfitting due to data scarcity, Fujioka et al. (5) and Pan et al. (6) started to use generative adversarial networks to simulate and enhance breast ultrasound sample data. The synthesized images will be further used for the training of convolutional neural networks. The semi-automatic classification model proposed by Bocchi et al. (7) is an outstanding early work to study breast lesion classification based on ultrasound video sequence data. In their proposed method, each imaging frame of the video is independently classified as benign or malignant after semi-automatic segmentation and morphological feature extraction. Subsequently, the classification results of all frames of the video are integrated to obtain reliable video-level results. This scheme results in a substantial improvement in the correct classification rate compared with the results of a single-image frame. At the same time, the uncertainty of classification judgments for certain frames reflects the clinical situation that lesions may present different characteristic manifestations when viewed from different viewpoints.

### 2.2 Contrastive learning

Traditional supervised learning methods rely heavily on a large amount of labeled training data available. In addition to the expensive labeling cost, this approach is also vulnerable to generalization error, spurious correlations, adversarial attaches, etc. (8). More and more studies start to find new ways out and start to learn feature representation by self-supervised learning. Contrastive learning is a discriminative approach, which aims to group similar samples closer together and dissimilar samples as far away from each other as possible. For computer vision tasks, methods such as MoCo (9), SwAV (10), and SimCLR (11) have produced comparable results to the state-of-the-art supervised methods in ImageNet (12) dataset. He et al. (9) proposed the momentum contrast method for unsupervised visual representation learning, which trains visual representation by constructing dynamic dictionaries with queueing and moving average encoders to match with encoded queries encoder. Compared with the direct comparison of features in general contrast learning, Caron et al. (10) save computational overhead by clustering data and computing online for different enhancements of the same image. Chen et al. (11) save computational overhead by incremental image augmentation and by feature representation and introducing a learnable linear transformation between the feature representation and contrast loss, further substantially improving the quality of the learned feature.

### 2.3 Attention mechanism

In the field of image classification, the attention mechanism is used to extract key regions and recognize images by spatial invariance. The STN (Spatial Transformer Networks) proposed by Jaderberg et al. (13) effectively addresses the insensitivity of convolutional networks to different viewpoints of the same thing through the attention mechanism. Wang et al. (14) proposed the non-local model to apply the self-attention mechanism to the computer vision tasks. For an input feature image, each pixel value is derived from the weighted average of other pixel features. SENet (15) proposed the squeeze-and-excitation module, which enhances important channels and suppresses invalid channels by automatically learning the importance of different channel features, thus improving model accuracy and reducing computational effort and complexity. Woo et al. (16) propose CBAM (Convolutional Block Attention Module) based on SENet. It extends the attentional dimension from focusing on the channel dimension to the spatial dimension.

## 3 Method

**Figure 1:**
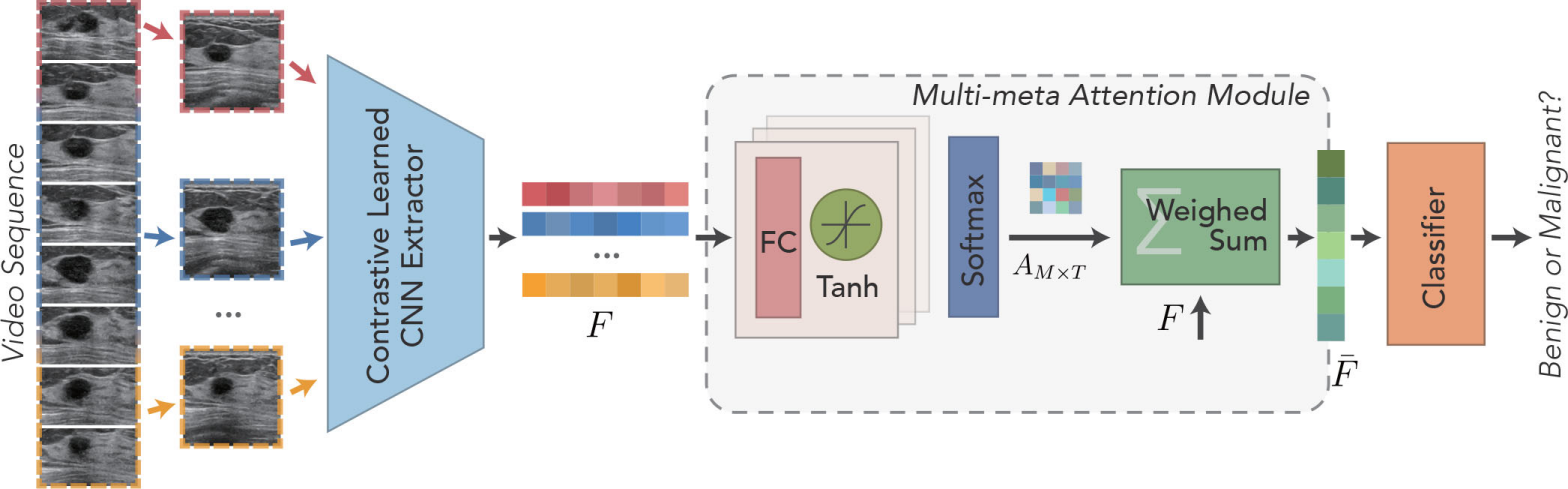
Schematic illustration of the developed Contrastive Learning guided Multi-meta Attention Network (CLMAN) for breast lesion classification in ultrasound sequence.

**Figure 1** shows the schematic illustration of the designed contrastive learning–guided multi-meta attention network (CLMAN). The network determines the input breast ultrasound sequence as benign or malignant, as well as the predicted score given to that. CLMAN consists of two main modules: a feature extraction module and a multi-meta attention module. The feature extraction module performs self-supervision training on the breast ultrasound video dataset by the contrast learning method before the formal training to the learn high-quality feature extraction patterns. For a given breast ultrasound sequence containing *T* frames, CLMAN first performs feature extraction on each frame by a pretrained feature extraction module to obtain independently encoded high-level feature vectors. Subsequently, the high-level feature vectors are aggregated for each frame in the multi-meta attention module. The module performs adaptive and fine-grained weight scoring along each feature dimension of each frame to form a compact and differentiated representation of breast lesions. Finally, the aggregated video-level feature vectors are used to determine the pathology of breast lesions by a linear classifier.

### 3.1 Deformed feature extraction module

**Figure 2:**
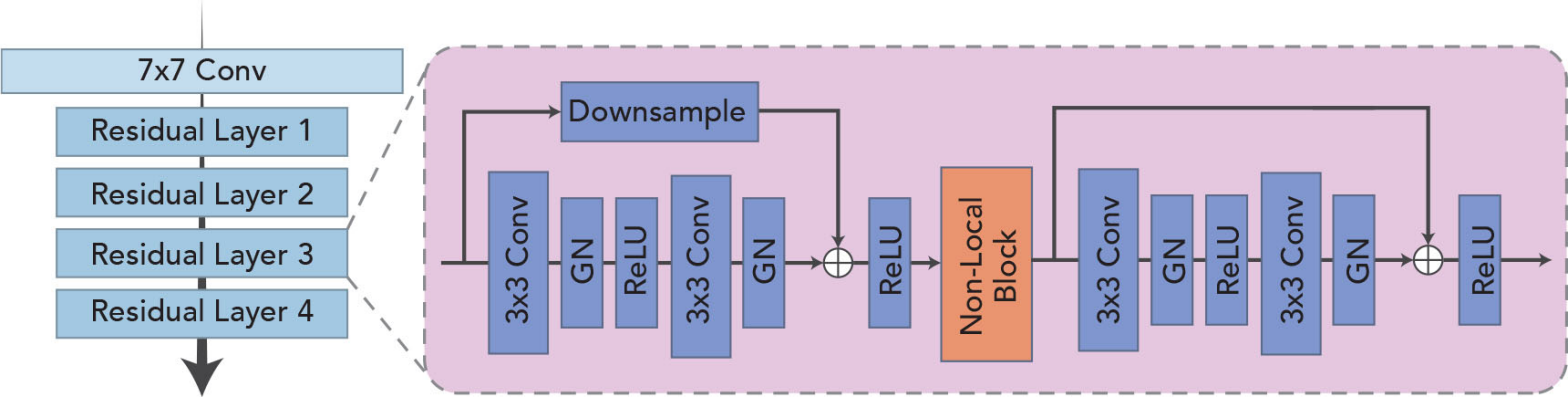
Schematic illustration of the deformed feature extraction module.

As shown in **Figure 2**, the feature extraction module is designed to extract features in a sequence and obtain a high-quality feature encoding vector for each frame, which is used for downstream tasks. The module is based on ResNet-18 (17) because the residual structure adopted effectively solves the problem of model degradation due to its depth, and the constant mapping also enhances the information transfer between the upper and lower layers. Because of the inherent multi-frame nature of a sequence, video classification tasks often take smaller batch sizes. Although the amount of training data per batch is sufficient in terms of the number of images, the general batch normalization approach may not be applicable when the model goes normalization because of the high similarity of pixel feature distribution across frames within the same video. In view of this, the group normalization (18) is used in each bottleneck structure in the basic feature extraction module to guarantee the stability of the distribution of the input features. For the problems of low quality and poor contrast of ultrasound imaging, it is especially important to focus on critical regions and suppress invalid regions effectively. The non-local (14) module is introduced and placed in the third and fourth stages of the feature extraction module for capturing spatially distant relationships. It focuses on the correlation between larger objects when the model level is shallow and pays more attention to the correlation between smaller objects when the model level is deep.

Suppose a breast ultrasound sequence *V* = {*v*_*t*_|*t*∈[0,*T*]} , where *v_t_* denotes the *t*th frame and *T* denotes the index of frames in the sequence. The feature extraction module Θ(·) extracts features from each frame to obtain the high-quality feature coding vector *F* = {*f*_*t*_|*t*∈[0,*T*]} for the whole sequence, which is given by


(1) $$f_{t}=\text{Θ}\left(v_{t}\right),\;t\in \left[0,T\right]$$

### 3.2 Contrast learning strategy

**Figure 3:**
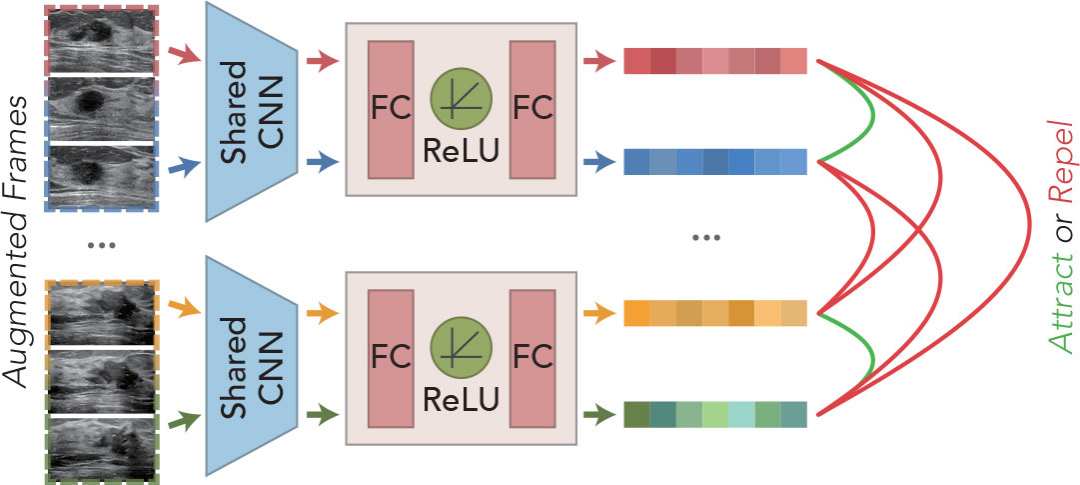
Schematic illustration of the predecessor task for the contrast learning strategy.

Breast ultrasound tumors tend to be characterized by large intraclass disparities and small interclass disparities in visual presentation. Moreover, the cross-sectional visualization of lesions presented at different stages within the same sequence often varies greatly. How to identify the diversity of different cross-sections of the same lesion is the basis for the correct classification of multi-frame sequences. Inspired by SimCLR (11), we borrowed this method of learning different data augmentation of the same image as positive samples together with negative samples composed of other images to train to determine the proximity of two features and applied it to video data, as shown in **Figure 3**.

For any *N* sequence clips, *M* frames are selected randomly as training samples, and then, the augmented training samples are extracted by the feature extraction network to obtain the high-level feature vectors. The extracted features are cascaded through MLP layers to obtain a tighter feature representation for the model to learn a better similarity representation. Assuming that the training samples provided for learning are $\left\{v_{m}^{n}|\forall_{n}\in \left[0,N\right],\;\forall_{m}\in \left[0,M\right]\right\}$ , the final feature representation can be obtained by the following:


(2) $${\overset{-}{f}}_{m}^{n}=\text{MLP}\left(\text{Θ}\left(\tau \left(v_{m}^{n}\right)\right)\right)$$

where *τ* denotes data augmentation. In the data augmentation stage, we mainly adopt the random combination of flip, crop, scale, modulation of brightness, contrast, and elastic transformation to increase the diversity of sample data.

### 3.3 Multi-meta attention module

The multi-meta attention module is applied to aggregate high-level feature vectors across frames of video to provide a compact and differentiated representation of mammary nodules. The module adaptively weighs all frames at a fine-grained level along each feature dimension, leveraging the valuable or discriminatory parts of each frame to facilitate commonality recognition without easily discarding or trivializing low-quality frames as the previous approaches have done. The feature extraction module trained by the contrast learning strategy is used to extract feature representations for each frame of the original sequence, denoted as follows:


(3) $$F^{t}={\left[\begin{array}{cccc} f_{1}^{t} & f_{2}^{t} & \cdots & f_{m}^{t} \end{array}\right]}_{m\times 1}^{T}$$

where *F^t^* denotes the *t*th frame feature vector with *m* dimensions.

As shown in **Figure 1**, a cascading attention module is applied to each frame feature to capture the attention representation better. Each attention module consists of a filter and an activation layer, which are cascaded to perform nonlinear feature learning:


(4) $$E_{l}^{t}=\sigma \left(W_{l}E_{l-1}^{t}+b_{l}\right)$$

where the fully connected layer is used as the filter and the Tanh function is used as the activation layer *σ*(·) for nonlinearly transformation. When l = 1, $E_{l-1}^{t}$ is defined as *F^t^*. For the obtained attention vectors of each frame, the attention linear weights corresponding to each of the *F^t^* channels are obtained by Softmax operation:


(5) $$A^{t}={\left[\begin{array}{c} \frac{exp\left(e_{1}^{t}\right)}{\sum_{j=1}^{T}exp\left(e_{1}^{j}\right)}\\ \frac{exp\left(e_{2}^{t}\right)}{\sum_{j=1}^{T}exp\left(e_{2}^{j}\right)}\\ \vdots \\ \frac{exp\left(e_{m}^{t}\right)}{\sum_{j=1}^{T}exp\left(e_{m}^{j}\right)} \end{array}\right]}_{m\times 1}$$

**Figure 4:**
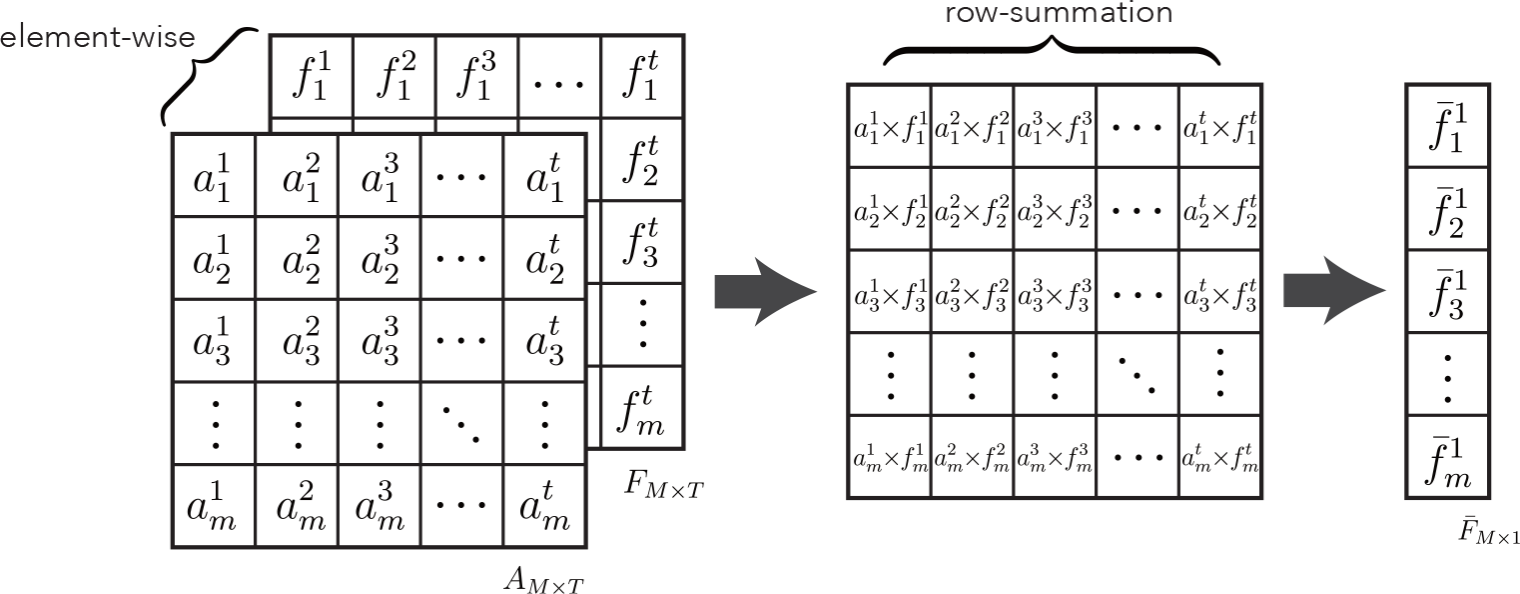
Schematic illustration of multi-meta attention operation .


The final aggregated feature is computed by multiplying the attention weights by the cumulative sum of the feature vectors, as shown in **Figure 4**. The specific aggregation operation can be expressed as follows:

(6) $$\overset{-}{F}=\sum_{t=1}^{T}A^{t}⊙F^{t}$$

where ⊙ denotes the matrix bitwise product. This aggregation module can weigh the importance of features at the dimensional level. Theoretically, it can achieve the best aggregation with good training. CLMAN uses a fair treatment of each frame of information to maximize the use of any of its valuable local features to facilitate the recognition of lesion sequence. Meanwhile, it is worth noting that the formula $\overset{-}{F}$ degrades to average pooling when each item in the attention matrix *A* is equal, and of course, the same formula also applies to maximum pooling in extreme cases.

In addition, using this module of mid-term aggregation of features allows the linear classifier to process sequence clips of arbitrary length, whereas the aggregation results $\overset{-}{F}$ have the same vector dimension as the individual features *F^t^* and the order remain constant, i.e., this aggregation module is insensitive to sequence order and temporal information and is generalizable to ultrasound sequence clips of arbitrary start and end points. The module’s parameters can be obtained by the standard backpropagation and gradient descent for supervised learning.

## 4 Experiments

### 4.1 Dataset

To evaluate the effectiveness of the developed network, we collected a dynamic breast ultrasound video sequences dataset with 268 videos, of which 152 sequences are malignant and 116 sequences are benign. All sequences are acquired by GE Healthcare equipment (Chicago, IL, USA), with L12-5 ultrasound probe and sampling frequency of 12 MHz, supported by the Xiamen University Xiang’an Hospital. A total of 107 of these sequences are randomly selected as the test set (about 40% of the total data volume), and the rest of the sequences are used as the training set. Data for both the training and test sets are obtained from cases of patients aged 20 years and older with definite benign or malignant pathological findings (BI-RADS categories 3 to 5) of breast lesions as determined by ultrasound.

### 4.2 Evaluation metrics

The six widely used metrics are utilized for quantitatively comparing different breast lesion ultrasound sequence classification methods. These are accuracy, average precision (AP), sensitivity, specificity, F_1_ score, and area under curve (AUC).

### 4.3 Implementation details

Our network is implemented on PyTorch (19) and trained using a SGD (Stochastic Gradient Descent) (20) with 320 epochs, an initial learning rate of 1 × 10^−4^, a momentum of 0.9, and a weight decay of 5 × 10^−4^. The sample length *T* is set to 16, whereas cross-entropy loss is set as the loss function. The whole architecture is trained on one GeForce RTX 2080 Ti GPU, and each GPU has a batch size of 8. In the contrast learning phase, NT-Xent (11) is used as the loss function, and the LARS (21) optimizer is used to train the model in the pre-task with 8,192 epochs, an initial learning rate of 9 × 10^−3^, and a weight decay of 1 × 10^−6^. The batch size here is set to 64. The learning rate is adjusted using the Cosine Annealing (22).

### 4.4 Ablation study

#### 4.4.1 Effectiveness of deformed extraction modules

We establish separate control groups based on ResNet-18 and compare the use of different components on the classification performance. As shown in **Table 1**, “ResNet18 (vanilla)” indicates the most primitive ResNet-18 architecture, “GN” denotes Group Norm, and “NL” denotes non-local module. To avoid the effect of the contrast learning strategy, none of the four settings in **Table 1** use that strategy. Compared with the plain ResNet-18 architecture, the feature extraction module with group norm has 4.68%, 6.33%, 1.92% and 3.18% improvement in accuracy, specificity, F1, and AUC, respectively. Meanwhile, the feature extraction module with the non-local module shows a steady increase in all six metrics, with 10.28% increase in accuracy, 6.4% increase in AP, 3.05% increase in sensitivity, 13.15% increase in specificity, 5.64% increase in F_1_, and 11.26% increase in AUC. The feature extraction module with the group norm and non-local module achieves the average best performance, with accuracy of 82.24%, AP of 81.16%, sensitivity of 82.08%, specificity of 82.35%, F_1_ of 85.50%, and AUC of 84.85%. It indicates that using the group norm and non-local module for the feature extraction module to obtain high-quality deep features has a certain facilitation effect.

**Table 1:** Quantitative comparisons for the effectiveness of deformed extraction modules.

Methods	Acc	AP	Sens	Spec	F_1_	AUC
ResNet18 (vanilla)	70.09	77.68	75.00	68.67	78.08	71.75
ResNet18 + GN	74.77	77.01	74.29	75.00	80.00	74.93
ResNet18 + NL	80.37	**84.08**	78.05	81.82	83.72	83.01
ResNet18 + GN + NL (ours)	**82.24**	81.16	**82.05**	**82.35**	**85.50**	**84.85**

“GN” denotes Group Norm, and “NL” denotes Non-local module. The bold values/numbers means that it is the largest among all the values at the column.

#### 4.4.2 Effectiveness of contrast learning strategy

The feature extraction module used by our network is pre-trained by a contrast learning strategy to effectively identify different geometric patterns of the same lesion under the same sequence imaging before formally training. **Table 2** verifies the impact of the contrast learning strategy, which is denoted as “CL”, on the model performance. The experiments show that the performance of the CLMAN decreases when the contrast learning strategy is removed. Specifically, accuracy, AP, sensitivity, specificity, F_1_, and AUC decreased by 6.55%, 11.62%, 12.39%, 3.57%, 5.54%, and 7.79%, respectively. It suggests that the contrast learning strategy can effectively alleviate the problem of large intraclass differences in the visual presentation of ultrasound lesions.

**Table 2:** Quantitative comparisons for the effectiveness of contrast learning strategy.

Methods	Acc	AP	Sens	Spec	F_1_	AUC
Without CL Guided	82.24	81.16	82.05	82.35	85.50	84.85
With CL (ours)	**88.79**	**92.78**	**94.44**	**85.92**	**91.04**	**92.64**

“CL” denotes the contrast learning strategy. The bold values/numbers means that it is the largest among all the values at the column.

#### 4.4.3 Effectiveness of multi-meta attention module

We conduct ablation experiments of multi-meta attention modules on the CLMAN model. First, the experiment considers the degenerate version of our multi-attention module, i.e., average pooling, as well as the extreme case of the maximum pooling and then compares them. Second, the LSTM (Long Short Term Memory) methods for long sequence feature capture are also compared in this experiment. In addition, we also compared attention modules proposed by other studies to demonstrate the advantage of the multi-meta attention module in video tasks. As shown in **Table 3**, “Multi-meta Att” denotes the multi-meta attention module, and “Average” and “Max-pooling” represent the degenerate average pooling and the extreme maximum pooling, respectively. According to **Table 3** the long sequence feature capture capability of LSTM is not fully applicable to ultrasound video imaging aggregation. The proposed classic attention modules that have often been effective in the past do not seem to be up to our video task. Meanwhile, the simple average pooling and maximum pooling methods achieved the best in terms of sensitivity or specificity, but the other metrics were not satisfactory. The proposed multi-meta attention scheme shows a 1.87% improvement in accuracy, 4.66% improvement in AP, 3.94% improvement in F_1_, and 5.12% improvement in AUC, with a stronger comprehensive capability. It indicates that the model has different fine-grained trade-offs for each part of the features, whereas such weights are learnable, and the simple and crude average pooling and maximum pooling approaches limit this adaptive capability.

**Table 3:** Quantitative comparisons for the effectiveness of multi-meta attention module.

Methods	Acc	AP	Sens	Spec	F_1_	AUC
With External Attention (23)	79.44	81.39	76.19	81.54	82.81	79.98
With Self-attention (24)	80.37	77.85	79.49	80.88	83.97	78.50
With Efficient Multi-head Self-attention (25)	80.37	78.51	81.08	80.00	84.21	77.49
With LSTM	83.18	77.91	96.43	78.48	87.32	79.47
With Average	86.92	85.26	**96.88**	82.67	89.86	87.48
With Max-pooling	85.05	88.12	80.43	**88.52**	87.10	87.52
With Multi-meta Attn (ours)	**88.79**	**92.78**	94.44	85.92	**91.04**	**92.64**

“Multi-meta Att” denotes the multi-meta attention module, and “Average” and “Max-pooling” represent the average pooling and the maximum pooling, respectively. The bold values/numbers means that it is the largest among all the values at the column.

### 4.5 Comparisons with state of the arts

To demonstrate the effectiveness and feasibility of the designed CLMAN model, **Table 4** selects from five papers nine existing methods commonly used to handle video classification task for comparison, including R3D (23), Times Former (27), MC3 (23), P3D (24), R(2 + 1)D (23), TIN(Res18, Res34, Res50)(29), and TSM (25). For providing a fair comparison, we obtain the classification results of all compared methods by exploiting their public implementations or by implementing them. We train these networks on our dataset and only set the batch size and epoch number to the same as ours.

**Table 4:** Quantitative comparisons of our network and compared methods on the collected ultrasound sequence dataset.

Methods	Acc	AP	Sens	Spec	F_1_	AUC
R3D (26)	75.70	80.03	82.14	73.42	81.69	77.56
Times Former (27)	77.57	71.34	77.78	77.46	82.09	72.08
MC3 (26)	77.57	80.83	81.25	76.00	82.61	78.86
P3D (28)	80.37	81.33	89.66	76.92	85.11	81.28
R(2+1)D (26)	82.24	87.36	90.32	78.95	86.33	84.85
TIN Res34 (29)	84.48	86.85	82.33	86.35	86.87	86.79
TIN Res50 (29)	85.05	85.53	88.94	83.10	88.06	86.66
TIN Res18 (29)	85.24	89.52	82.50	87.11	87.52	87.30
TSM (30)	86.92	89.72	91.67	84.51	89.55	90.22
CLMA-Net (ours)	**88.79**	**92.78**	**94.44**	**85.92**	**91.04**	**92.64**

The bold values/numbers means that it is the largest among all the values at the column.

CLMAN performs on par with the best of the methods compared and even better suited for video-level classification tasks of breast ultrasound sequence, with accuracy improved by 1.87%, AP improved by 3.06%, sensitivity improved by 2.77%, specificity by 1.44%, F_1_ improved by 1.49%, and AUC improved by 2.42%.

**Figure 5:**
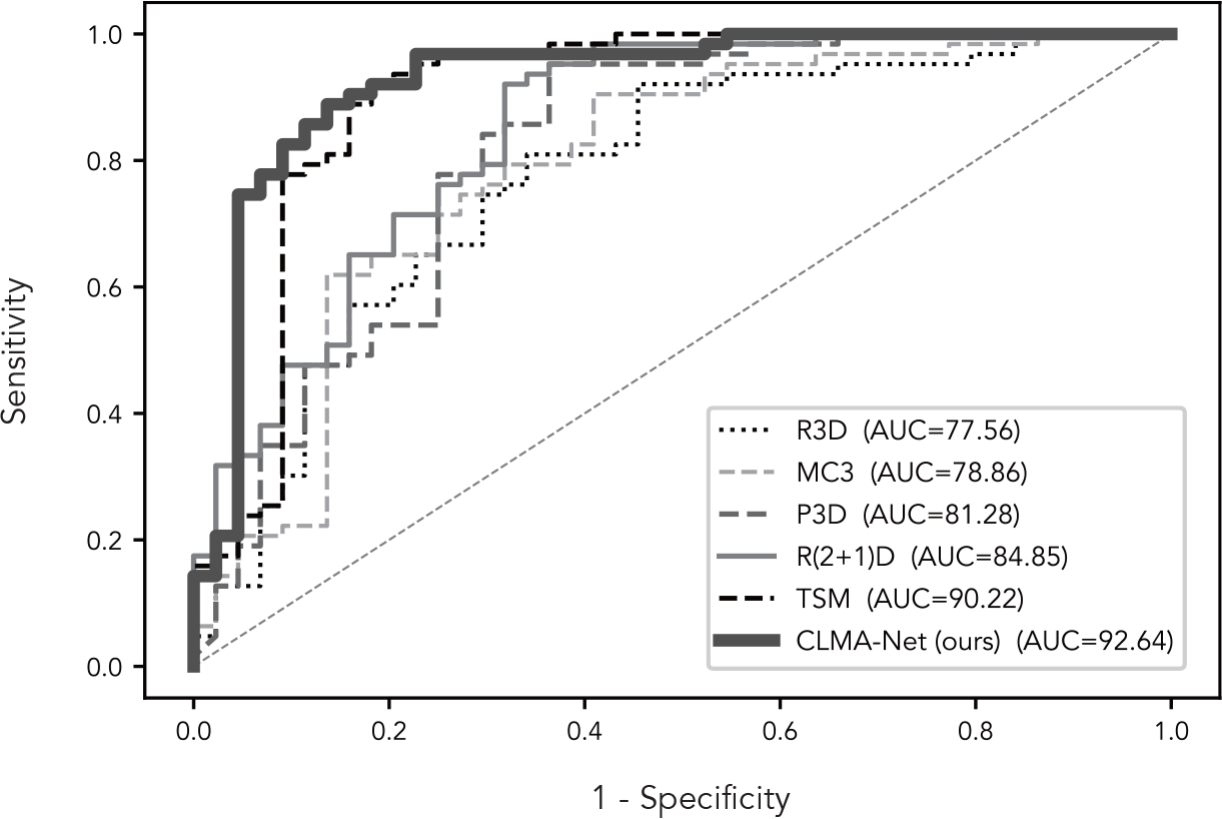
ROC curves of our network and compared methods.

More visually, **Figure 5** shows the ROC curves of CLMAN with the above five of the nine methods. The performance of R3D, MC3, P3D, and R(2 + 1)D is similar, and the AUC remains around 80%, whereas the area of TSM and CLMAN is comparable, both exceeding 90%.

## 5 Conclusion

In this paper, we first collected 268 video sequences constituting a video dataset for breast ultrasound classification. Moreover, we propose a CLMAN for lesion diagnosis of ultrasound breast sequences in arbitrary length. Our approach is able to learn the attention weights of each feature dimension adaptively and autonomously in both spatial and temporal dimensions while using a contrast learning predecessor task to effectively address several challenges of the ultrasound video sequence classification problem. Experimental results on the collected dataset show that our network achieves superior diagnostic performance for breast lesions than the state-of-the-art video classification methods.

## Data availability statement

The raw data supporting the conclusions of this article will be made available by the authors, without undue reservation.

## Author contributions

XH and SH participated in the design of the study. XH and SH collected the data. ZL and XH performed the statistical analysis. ZL and XH wrote the manuscript. ZL, M-TC, and LW revised the manuscript. FW makes its contributions to prepare and revise the response letter for revised paper,and provide a research grant to cover the publication fee. All authors contributed to the article and agreed to the submitted version.

## Acknowledgments

This work was supported by the Research Grants Council of Hong Kong (Project: UGC/FDS16/M08/18).

## Conflict of interest

The authors declare that the research was conducted in the absence of any commercial or financial relationships that could be construed as a potential conflict of interest.

## Publisher’s note

All claims expressed in this article are solely those of the authors and do not necessarily represent those of their affiliated organizations, or those of the publisher, the editors and the reviewers. Any product that may be evaluated in this article, or claim that may be made by its manufacturer, is not guaranteed or endorsed by the publisher.
